# Supplementary material for: Targeted Sequencing of Pancreatic Adenocarcinomas from Patients with Metachronous Pulmonary Metastases
Source: Genes (Basel). 2020 Nov 24;11(12):1391. doi: 10.3390/genes11121391 (PMC7760784; doi:10.3390/genes11121391)
Supplement: Supplementary file 1 [file genes-11-01391-s001.zip › Hlavac et al Supplementary Figures 20-11-24VH.pdf]

Supplementary Figures

Targeted Sequencing of Pancreatic Adenocarcinomas from Patients with Metachronous Pulmonary Metastases

Viktor Hlavac[1,2], Beatrice Mohelnikova-Duchonova[3], Martin Lovecek[4], Jiri Ehrmann[5], Veronika Brynychova[1,2], Katerina Kolarova[3] and Pavel Soucek[1,2]

[1]*Biomedical Center, Faculty of Medicine in Pilsen, Charles University, Pilsen, Czech Republic;*

[2]*Toxicogenomics Unit, National Institute of Public Health, Prague, Czech Republic;*

[3]*Department of Oncology, Institute of Molecular and Translational Medicine, Faculty of Medicine and Dentistry, Palacky University, Olomouc, Czech Republic;*

[4]*Department of Surgery I, University Hospital Olomouc and Faculty of Medicine and Dentistry, Palacky University, Olomouc, Czech Republic;*

[5]*Department of Clinical and Molecular Pathology, University Hospital Olomouc and Faculty of Medicine and Dentistry, Palacky University, Olomouc, Czech Republic.*

Address for correspondence: Viktor Hlavac, PhD, Laboratory of Pharmacogenomics, Biomedical Center, Faculty of Medicine in Pilsen, Charles University, alej Svobody 76, 323 00 Pilsen, Czech Republic, phone/fax+420-377-593-840; e-mail: viktor.hlavac@lfp.cuni.cz; http://www.biomedic-plzen.cz/en

Supplementary Figure S1: IGV comparison of sequencing and ddPCR results showing reads with uncalled alternative bases

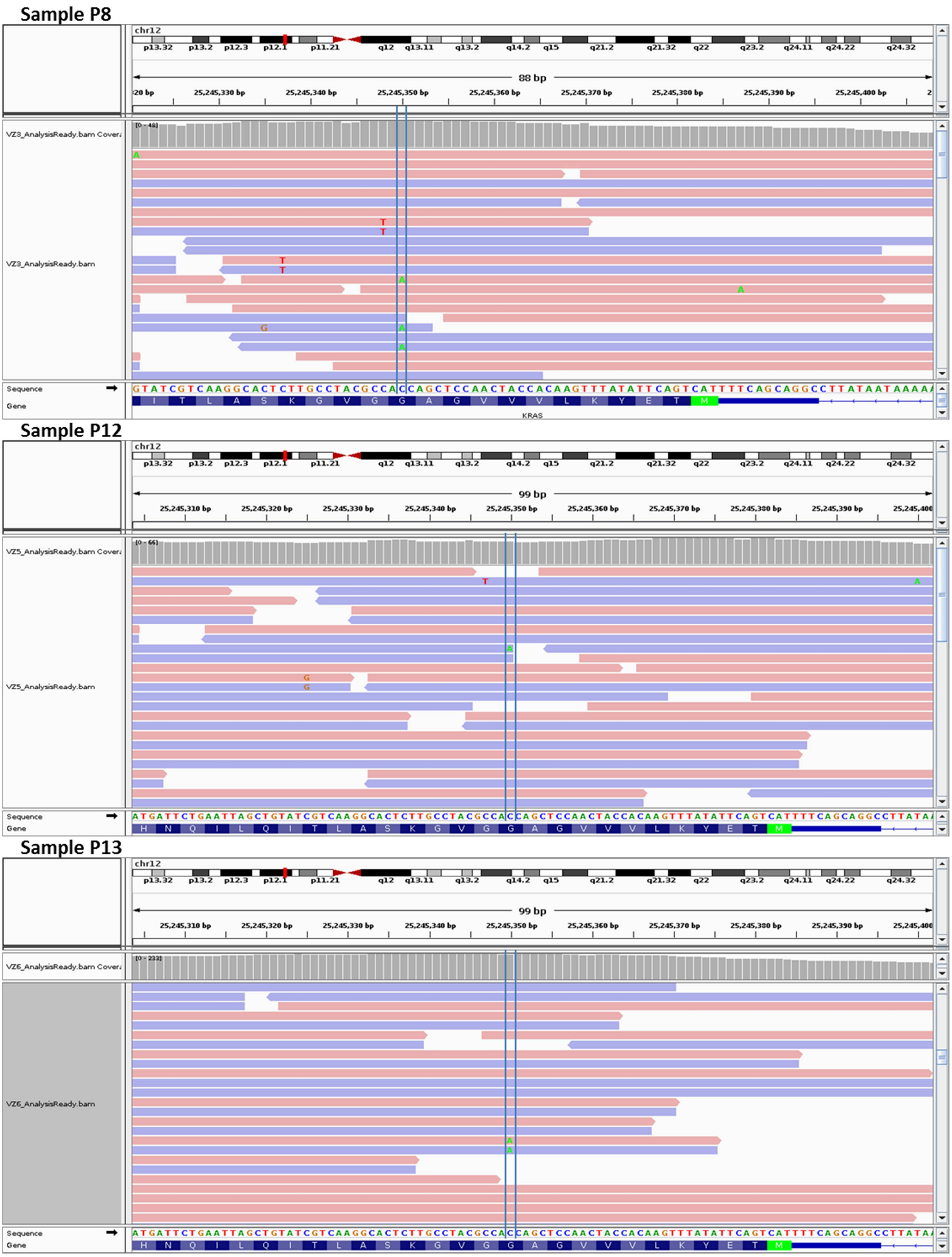

Supplementary Figure S2: Oncoplot of primary tumors depicting genes significantly mutated in Broad Institute PDAC (A) and LUAD (B) cohorts

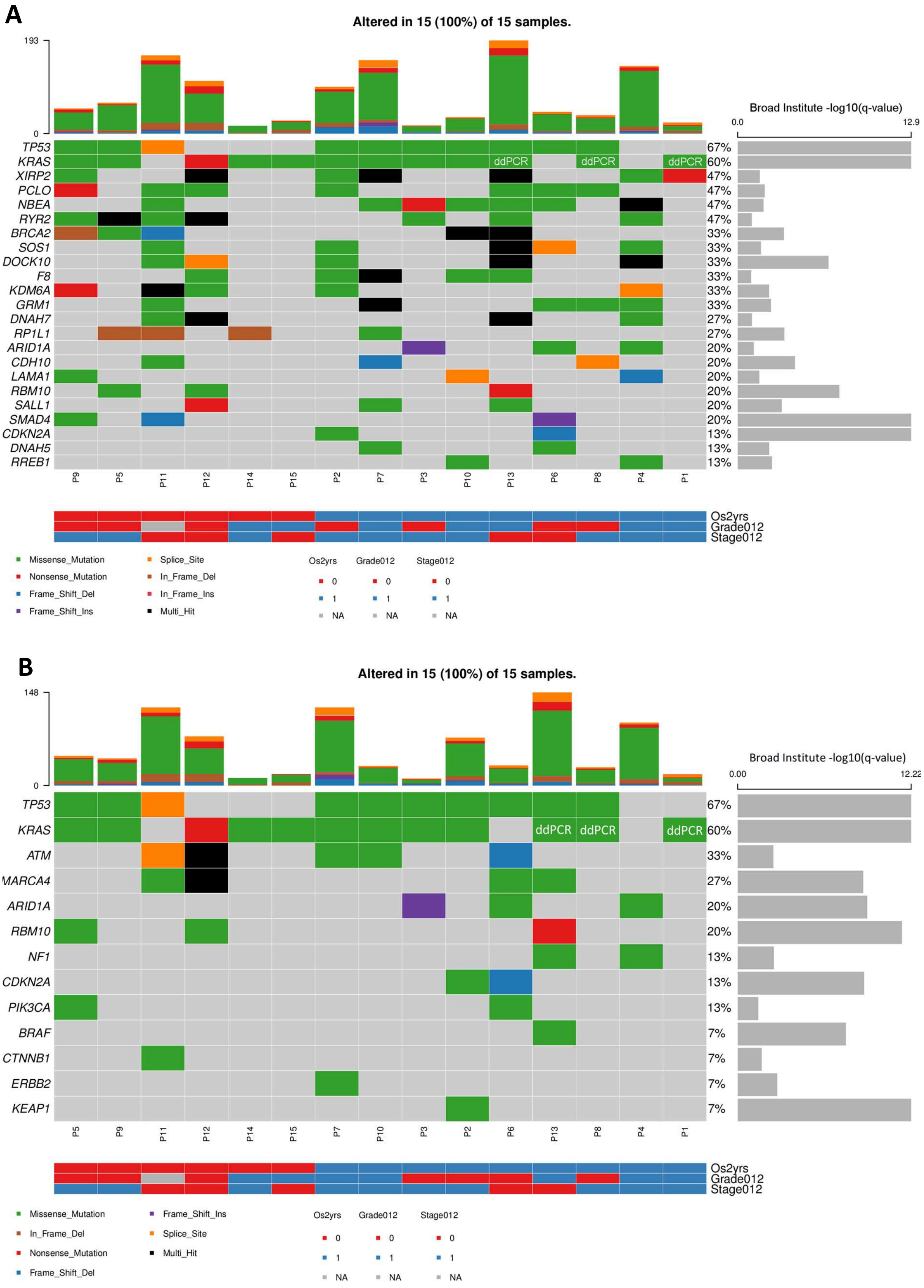

Supplementary Figure S3: The summary of the distribution of variants in three sample pairs of primary tumors and matched pulmonary metastases

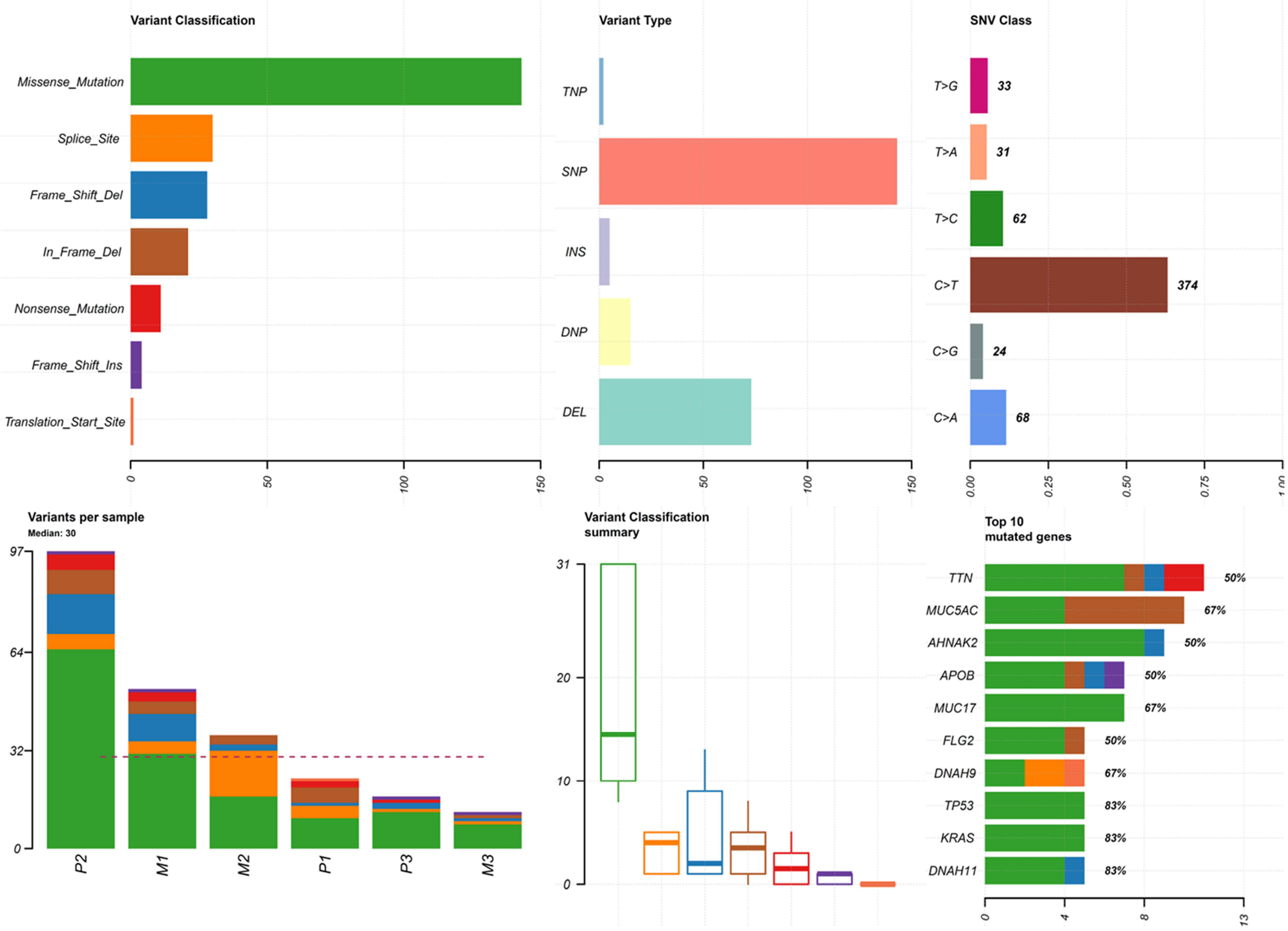

Supplementary Figure S4: Oncoplot of comparison between primary tumors and matched pulmonary metastases depicting genes significantly mutated in Broad Institute PDAC (A) and LUAD (B) cohorts

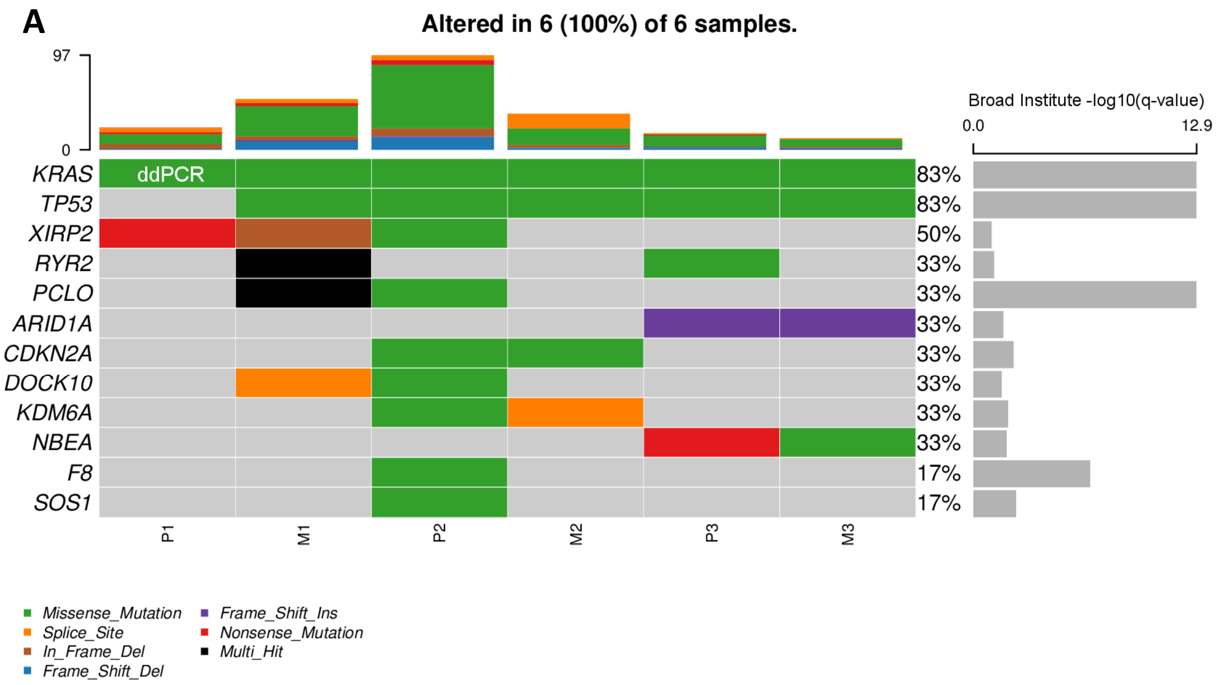

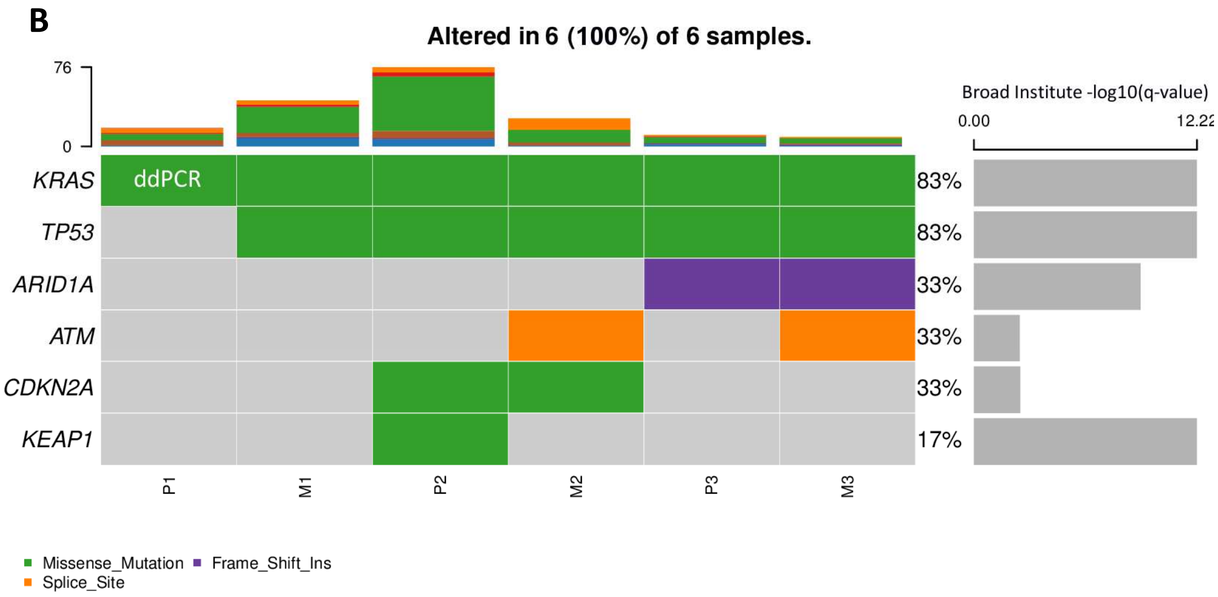

Supplementary Figure S5: Overall survival plot of available TCGA datasets stratified by KRAS and TP53 mutation status (cBioPortal, refs. [40,41])

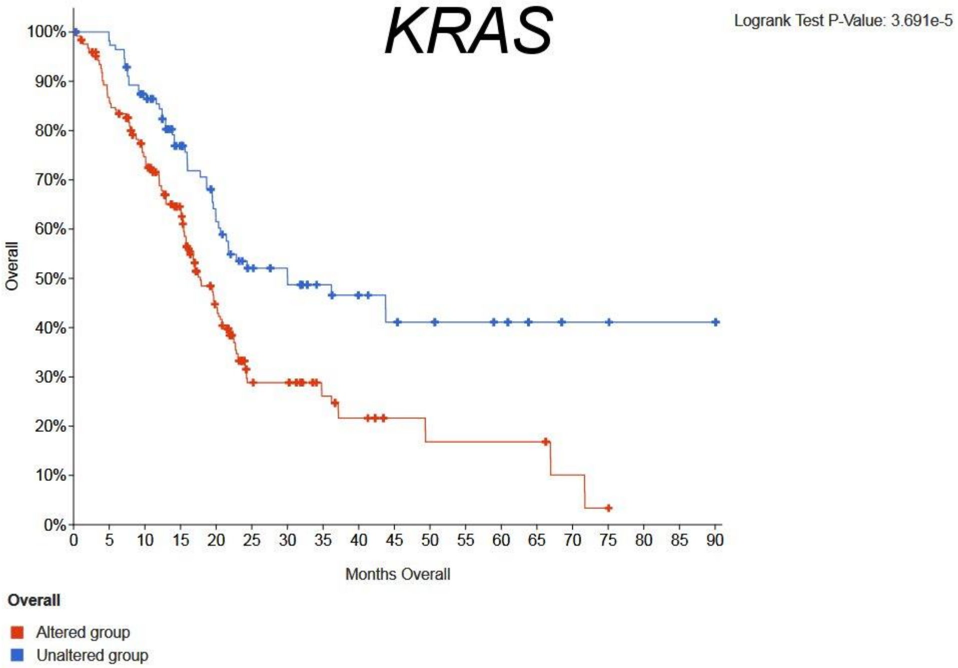

	Number of Cases, Total	Number of Cases, Deceased	Median Months Overall
Altered group	254	151	17.79
Unaltered group	114	48	30.02

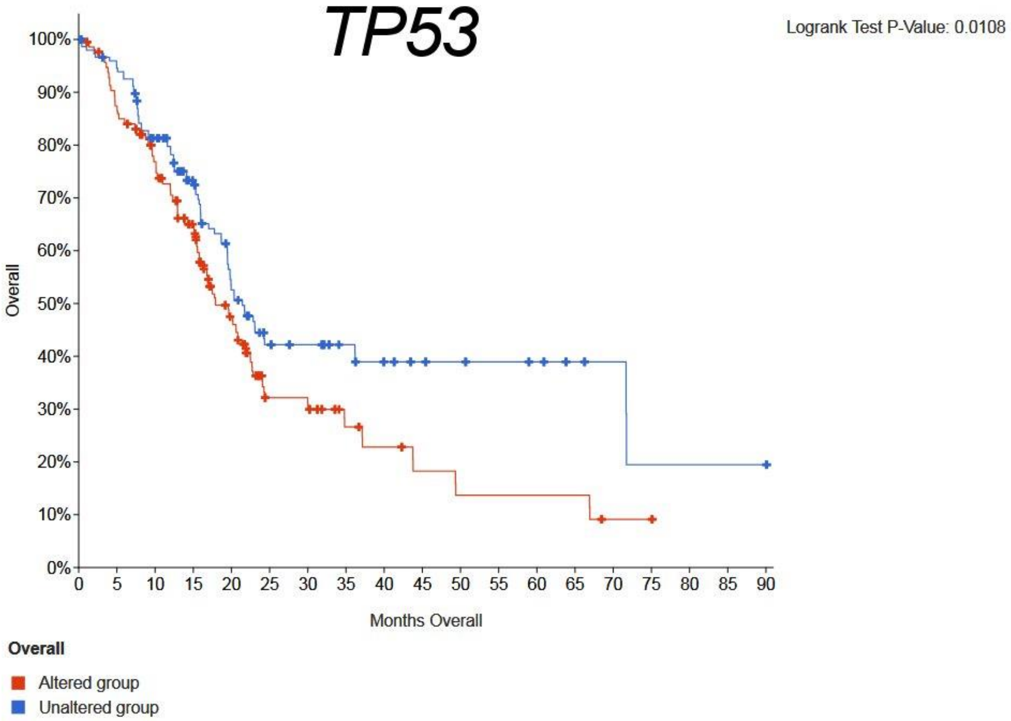

	Number of Cases, Total	Number of Cases, Deceased	Median Months Overall
Altered group	212	126	17.92
Unaltered group	156	73	21.44
